# In search of druggable targets for GBM amino acid metabolism

**DOI:** 10.1186/s12885-017-3148-1

**Published:** 2017-02-28

**Authors:** Eduard H. Panosyan, Henry J. Lin, Jan Koster, Joseph L. Lasky

**Affiliations:** 1 Los Angeles Biomedical Research Institute and Department of Pediatrics at Harbor-UCLA Medical Center, Box 468, 1000 W. Carson Street, N25, Torrance, CA 90509 USA; 2 Department of Oncogenomics, Academic Medical Center of the University of Amsterdam, Amsterdam, The Netherlands

**Keywords:** Glioblastoma (GBM), Amino-acid (AA) metabolism, BCAT1 (branched chain amino acid transaminase 1), Asparagine (Asn), Glutamine (Gln)

## Abstract

**Background:**

Amino acid (AA) pathways may contain druggable targets for glioblastoma (GBM). Literature reviews and GBM database (http://r2.amc.nl) analyses were carried out to screen for such targets among 95 AA related enzymes.

**Methods:**

First, we identified the genes that were differentially expressed in GBMs (3 datasets) compared to non-GBM brain tissues (5 datasets), or were associated with survival differences. Further, protein expression for these enzymes was also analyzed in high grade gliomas (HGGs) (*proteinatlas.org*). Finally, AA enzyme and gene expression were compared among the 4 TCGA (The Cancer Genome Atlas) subtypes of GBMs.

**Results:**

We detected differences in enzymes involved in glutamate and urea cycle metabolism in GBM. For example, expression levels of BCAT1 (branched chain amino acid transferase 1) and ASL (argininosuccinate lyase) were high, but ASS1 (argininosuccinate synthase 1) was low in GBM. Proneural and neural TCGA subtypes had low expression of all three. High expression of all three correlated with worse outcome. ASL and ASS1 protein levels were mostly undetected in high grade gliomas, whereas BCAT1 was high. GSS (glutathione synthetase) was not differentially expressed, but higher levels were linked to poor progression free survival. ASPA (aspartoacylase) and GOT1 (glutamic-oxaloacetic transaminase 1) had lower expression in GBM (associated with poor outcomes). All three GABA related genes -- glutamate decarboxylase 1 (GAD1) and 2 (GAD2) and 4-aminobutyrate aminotransferase (ABAT) -- were lower in mesenchymal tumors, which in contrast showed higher IDO1 (indoleamine 2, 3-dioxygenase 1) and TDO2 (tryptophan 2, 3-diaxygenase). Expression of PRODH (proline dehydrogenase), a putative tumor suppressor, was lower in GBM. Higher levels predicted poor survival.

**Conclusions:**

Several AA-metabolizing enzymes that are higher in GBM, are also linked to poor outcome (such as BCAT1), which makes them potential targets for therapeutic inhibition. Moreover, existing drugs that deplete asparagine and arginine may be effective against brain tumors, and should be studied in conjunction with chemotherapy. Last, AA metabolism is heterogeneous in TCGA subtypes of GBM (as well as medulloblastomas and other pediatric tumors), which may translate to variable responses to AA targeted therapies.

**Electronic supplementary material:**

The online version of this article (doi:10.1186/s12885-017-3148-1) contains supplementary material, which is available to authorized users.

## Background

In addition to surgery and radiation, brain tumors are subject to systemic therapies, which circulate in the bloodstream and affect cancer cells all over the body. The systemic therapies for cancer can be grouped into 4 main categories: (1) DNA damaging and/or repair suppressing agents [1] (e.g., cytotoxic chemotherapy); (2) cell signaling inhibition [1–3] (e.g., blocking tumor angiogenesis and tyrosine kinases); (3) immunotherapy [4, 5]; and (4) metabolic strategies [6]. Metabolic approaches are based on assumed differences in metabolism in cancer cells compared to normal tissues [6, 7]. Antimetabolites largely act by diminishing synthesis of molecules essential for cancer cell survival, either by substrate depletion or by interfering with enzyme (s) [6]. Classic examples include asparaginase for acute leukemias [8] and the anti-folate drug, methotrexate, for a variety of tumors [9]. A major advantage of antimetabolites is the absence of direct DNA damage, which leads to significant bone marrow toxicity [10], and may cause secondary malignancies [11]. Although signaling inhibition and immunotherapy also lack myelosuppression, clinical efficacy of these “targeted” strategies has been limited to only certain types of cancer [3, 5].

The recent discovery of mutations in IDH (isocitrate dehydrogenase, a Krebs cycle enzyme) in some gliomas [12] has renewed interest in antimetabolic approaches in neuro-oncology [13]. In addition to the use of IDH1 and IDH2 inhibitors [12], targeting lipid [14] and carbohydrate (i.e., energy) metabolism has also been an area of research (e.g., use of metformin [15]). Moreover, the augmented amino acid metabolism in brain tumors has led to enhanced neuro-imaging with radiolabeled amino acids as a diagnostic tool [16, 17]. However, manipulation of amino acid metabolism remains an under-studied topic in current neuro-oncology research, and is therefore the topic of this investigation.

## Methods

Publically available databases and published literature were used for this study. Our general hypotheses were: (a) differential expression of genes related to amino-acid (AA) metabolism and the corresponding enzymes can help to identify potential drug targets for glioblastoma treatment; (b) correlations among certain genes (or enzymes) and patient survival may indicate clinical relevance; and (c) subtypes of brain tumors may show heterogeneity in AA metabolism.

First, we constructed a list of 95 genes that code for amino-acid metabolizing enzymes, based on known biochemical pathways (Table 1) [18]. Analyses of 22 AA KEGG (Kyoto Encyclopedia of Genes and Genomes) pathways suggested by TCGA data were also used in developing the list. To assess potential differential expression, we used the “R2: Genomic Analysis and Visualization Platform” database (s) at http://r2.amc.nl [19]. R2 contains multiple datasets on various pathological conditions from gene expression microarrays. Datasets generated on 2 Affymetrix chip types, both analyzed by MAS5.0, were used in our study. In addition, certain datasets allowed patient survival analysis in relation to gene expression levels. Selected glioblastoma (GBM) datasets in R2 also allowed analysis based on TCGA subtypes.

**Table 1:** Ninety-five genes for amino acid metabolism related enzymes that were subjected to initial screening

Pathways	Gene/Enzyme
Alanine, asparagine, aspartate, glutamine, & glutamate metabolism:	1. ABAT: 4-aminobutyrate aminotransferase 2. ADSL: adenylosuccinate lyase 3. ADSS: adenylosuccinate synthetase 4. AGXT: alanine-glyoxylate aminotransferase 5. DDO: D-aspartate oxidase 6. ASNS: aspargine synthetase 7. ASPA: aspartoacylase 8. GAD1: glutamate decarboxylase 1 9. GAD2: glutamate decarboxylase 2 10. GOT1: glutamic-oxaloacetic transaminase 1, soluble (i.e., AST: aspartate transaminase or aminotransferase, AspAT/ASAT/AAT or SGOT) 11. GOT2: glutamic-oxaloacetic transaminase 2, mitochondrial 12. GPT: glutamic-pyruvate transaminase (i.e. ALT: alanine aminotransferase) 13. GLUD1: glutamate dehydrogenase 1 14. GLUD2: glutamate dehydrogenase 2 15. ALDH5A1: Aldehyde Dehydrogenase 5 Family, Member A1 16. GLUL: glutamine synthetase (i.e., GS) 17. GFPT2: glutamine-fructose-6-phosphate transaminase 2 18. MECP2: methyl CpG binding protein 2 19. GLS: glutaminase
Histidine metabolism:	20. ALDH1B1: aldehyde dehydrogenase 1 family, member B1 21. CNDP2: CNDP dipeptidase 2 (metallopeptidase M20 family) 22. HDC: Histidine dexarboxylase 23. HAL: histidine ammonia-lyase (i.e., Histidase: HIS or HSTD)
Leucine, isoleucine, & valine metabolism:	24. BCAT1: branched chain amino-acid transaminase 1, cytosolic 25. BCAT2: branched chain amino-acid transaminase 2, mitochondrial 26. LRS: Leucyl-tRNA synthetase 27. BCKDHB: branched chain keto acid dehydrogenase E1, beta polypeptide 28. ILVBL: ilvB (bacterial acetolactate synthase)-like 29. PCCB: propionyl CoA carboxylase, beta polypeptide
Lysine metabolism:	30. AASDHPPT: L-aminoadipate-semialdehyde dehydrogenase-phosphopantetheinyl transferase 31. PIPOX: pipecolic acid oxidase 32. WHSC1L1: Wolf-Hirschhorn syndrome candidate 1-like 1
Phenylalanine metabolism:	33. PAH: phenylalanine hydroxylase 34. FAH: fumarylacetoacetate hydrolase (fumarylacetoacetase)
Serine, glycine, & threonine metabolism:	35. ALAS1: 5′-aminolevulinate synthase 1 36. ALAS2: 5′-aminolevulinate synthase 2 37. GCAT: glycine C-acetyltransferase 38. PHGDH: phosphoglycerate dehydrogenase 39. PSAT1: phosphoserine aminotransferase 1 40. PSPH: phosphoserine phosphatase 41. SDS: serine dehydratase 42. SHMT1: serine hydroxymethyltransferase 1 43. SHMT2: serine hydroxymethyltransferase 2 44. SPTLC1: serine palmitoyltransferase, long chain base subunit 1 45. SPTLC2: serine palmitoyltransferase, long chain base subunit 2 46. SPTLC3: serine palmitoyltransferase, long chain base subunit 3 47. PPP2R4: protein phosphatase 2A activator, regulatory subunit 4 (i.e., PP2A) 48. ALAD: Aminolevulinic dehydrase
Tyrosine metabolism:	49. PNMT: phenylethanolamine N-methyltransferase 50. TH: tyrosine hydroxylase 51. TAT: tyrosine aminotransferase 52. DDC: DOPA decarboxylase (aromatic L-amino acid decarboxylase)
Cysteine, methionine, & glutathione metabolism:	53. CCBL1: cysteine conjugate-beta lyase, cytoplasmic 54. CCBL2: cysteine conjugate-beta lyase 2 55. LDHA: lactate dehydrogenase A 56. AHCY: adenosylhomocysteinase 57. MDH2: malate dehydrogenase 2, NAD (mitochondrial) 58. TYMS: thymidylate synthase 59. CTH: cystathionine gamma-lyase 60. GCLC: glutamate-cysteine ligase, catalytic subunit 61. GCLM: glutamate-cysteine ligase, modifier subunit 62. GSS: Glutathione synthetase 63. MTR: 5-methyltetrahydrofolate-homocysteine methyltransferase 64. MAT2A: methionine adenosyltransferase II, alpha
Arginine and proline metabolism:	65. OAT: ornithine aminotransferase 66. CKM: creatine kinase, muscle 67. LAP3: leucine aminopeptidase 3 68. ASL: argininosuccinate lyase 69. ASS1: argininosuccinate synthetase 1 70. ADC: arginine decarboxylase 71. DDAH2: dimethylarginine dimethylaminohydrolase 2 72. GATM: glycine amidinotransferase (L-arginine:glycine amidinotransferase) (i.e., AGAT: arginine:glycine amidinotransferase) 73. ARG1: arginase 1 74. PADI2: peptidyl arginine deiminase, type II 75. PYCR1: pyrroline-5-carboxylate reductase 1 76. PRODH: proline dehydrogenase (oxidase) 1
Tryptophan metabolism:	77. AANAT: aralkylamine N-acetyltransferase 78. TDO2: tryptophan 2,3-dioxygenase 79. TPH1: Tryptophan hydroxylase 1 80. IDO1: indoleamine 2,3-dioxygenase 1
Selenocompound metabolism:	81. MARS: methionyl-tRNA synthetase 82. SEPHS1: selenophosphate synthetase 1
Other:	83. AADAT: aminoadipate aminotransferase 84. UROS: Uroporphyrineogen synthase 85. UROD: uroporphyrinogen decarboxylase 86. CPS1: carbamoyl-phosphatesynthase 1, mitochondrial 87. OTC: ornithine carbamoyltransferase 88. PDXP: pyridoxal (pyridoxine, vitamin B6) phosphatase 89. PNPO: pyridoxamine 5′-phosphate oxidase
Amino acid transporters:	90. SLC3A2: solute carrier family 3 (amino acid transporter heavy chain), member 2 (i.e., 4F2hc) 91. SLC7A11: solute carrier family 7 (anionic amino acid transporter light chain, xc- system), member 11 (i.e., xCT) 92. SLC7A7 solute carrier family 7 (amino acid transporter light chain, y + L system), member 7 (i.e., LAT3) 93. SLC7A5: solute carrier family 7 (amino acid transporter light chain, L system), member 5 (i.e., LAT1) 94. SLC1A5: solute carrier family 1 (neutral amino acid transporter), member 5 (i.e., ASCT2) 95. SLC6A14: solute carrier family 6 (amino acid transporter), member 14

Eight datasets, including 3 with GBM and 5 with non-GBM brain tissues, were used to review metabolic differences in GBM (Table 2). In order to minimize ambiguity, we selected 5 non-GBM/control datasets containing information on non-neoplastic brain tissues with or without concomitant conditions (such as mild cognitive impairment, agonal stress or Parkinson’s disease). Initially, we screened the entire pool of 95 genes in 3 of the largest GBM datasets, using R2 bar-graphing tools and Kaplan-Meier curves to identify potentially relevant candidates (representative graphs are shown in Results). Gene probes were selected based on higher expression and availability of the same probe across the datasets and for Kaplan-Meier analysis. About a third of the genes appeared to be either differentially expressed, or have significant association with clinical outcome (i.e., progression free survival and/or overall survival). A few genes were included in our analysis solely based on literature reports on relevance to GBM. For the 34 genes resulting from this initial analysis, we aimed to verify quantitative expression in GBMs and compare these values to expression levels in non-GBM brain tissues.

**Table 2:** Five brain tumor (3 GBM) and five non-brain tumor datasets used

#	Name of dataset	Number of samples	Platform - Chiptype
1	Normal Brain regions - Berchtold	172	u133p2
2	Normal Brain PFC – Harris	44	u133p2
3	Disease^a^ Brain - Liang	34	u133p2
4	Tumor Glioblastoma - Loeffler	70	u133p2
5	Tumor Glioblastoma - Hegi	84 (80 tumors)	u133p2
6	Normal Brain agonal stress - Li	1168	u133a
7	Disease Brain Parkinson - Moran	47	u133a
8	Tumor Glioblastoma - TCGA	540	u133a
9	Mixed Pediatric Brain (Normal-Tumor) – Donson	130 (117 tumors)	u133p2
10	Tumor Medulloblastoma – Gilbertson	76 (73 tumors)	u133p2

^a^Brain tissues are from individuals who had been diagnosed with mild cognitive impairment. Detailed description of each dataset is available at http://r2.amc.nl

### Statistics for differential gene expression in GBM versus non-GBM

Datasets 1–5 from Table 2 were generated by Affymetrix Human Genome U133 Plus 2.0 arrays (u133p2), and datasets 6–8 by u133pa. To avoid possible misinterpretation of results due to use of the two different arrays, the average gene expression levels were kept in two groups: Mean-A (for datasets 1, 2 and 3); and Mean-B (for datasets 6 and 7). Next, for each gene we calculated 3 ratios of expression, from 3 GBM datasets (using GBM/non-GBM from the same array):

1. Ratio 1 = Gene expression from dataset #4 over Mean-A,
2. Ratio 2 = Gene expression from dataset #5 over Mean-A, and
3. Ratio 3 = Gene expression from dataset #8 over Mean-B.

**Fig. 1:**
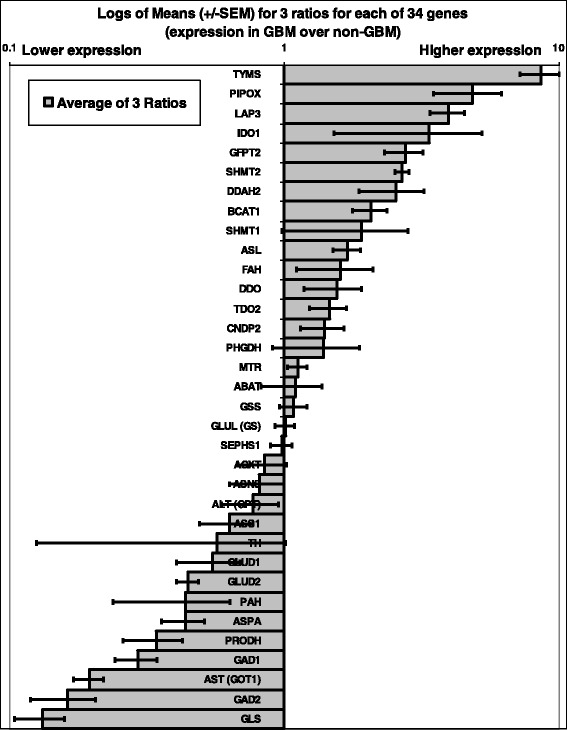
Differential expression of 34 genes in glioblastoma (GBM). The *x-axis* represents the logarithm of the ratio of gene expression in GBM over expression in normal brain tissue (calculations as described in Methods). Each horizontal bar with errors represents a gene, and ratios are shown as means ± standard errors. Genes listed starting from TDO2 and above are over-expressed genes. Genes listed starting from ASS1 and below are under-expressed genes. Refer to Table 1 for abbreviations. Log of Mean values = 1 indicates equal expression in GBMs and normal brain tissue

Last, averages (± standard errors) of ratios 1, 2 and 3 were calculated for each gene (Fig. 1). This procedure allowed us to evaluate differential expression more reliably, and to eliminate a few genes that were proposed in the initial screen.

### Protein expression of AA related enzymes in high grade gliomas

**Fig. 2:**
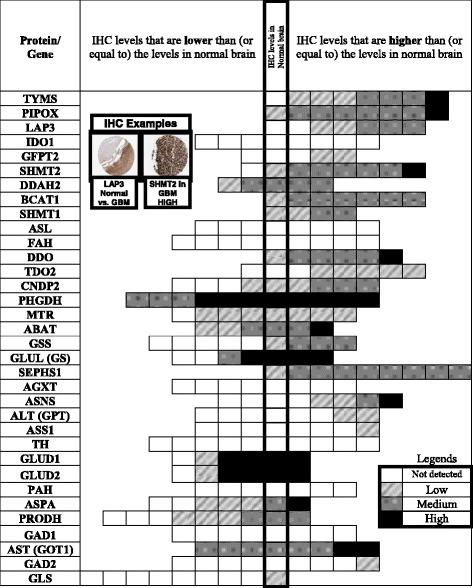
Detection of 34 proteins by immunohistochemistry (IHC) in high grade gliomas. The *left side* represents proteins expressed at levels less than or equal to levels in normal brain tissue. The *right side* shows IHC data for proteins expressed at levels greater than levels in normal brain tissue. The x-axis corresponds to the number of samples for each gene. *Color codes* indicate the intensity of protein expression for a given gene (as shown on legend). For example, for PHDGH (phosphoglycerate dehydrogenase) there were 11 samples -- 8 showed high expression, and 3 showed low expression. *Left upper illustration* exemplifies IHCs for couple of proteins. Abbreviations are as in Table 1. There is less overt clustering in the *right upper* and *left lower quadrants* compared to results in Fig. 1, because gene and protein over-expression match only in part

Gene expression levels may not always correlate with protein production. Therefore, further verification of our findings at the protein level was considered. An online database (Proteinatlas.org) contains immunohistochemical (IHC) data on most human proteins in a variety of tissues, including gliomas, as part of a cancer atlas project [20]. The database was used to evaluate protein expression for the panel of 34 genes with possible differential expression in high grade gliomas (HGGs). Each tested tumor has a semi-quantitative antibody staining score (i.e., high, medium, low or not detected; representative examples are shown in Fig. 2). The average number of high grade glioma specimens tested for each protein was 8 (range, 5–11). Figure 2 shows the numbers of tumors with each of the 4 levels of antibody staining, for a given protein. IHC for a few proteins was done with more than one antibody. Selection was based on the most consistent staining pattern, for these proteins.

### TCGA database in R2: subtypes and survival analyses

This enriched database contains 540 GBM samples and is the largest among the 3 tested. It allows detailed analysis of patient survival with the Kaplan-Meier method. Comparison of expression of various genes among the 4 TCGA subtypes is also possible (proneural, neural, classical and mesenchymal; 85 specimens). For Kaplan-Meier analysis, both progression-free survival (PFS) and overall survival (OS) were assessed for each of the genes with various cut-offs, aiming for *P* values <0.05 (which were considered significant). However, survival analysis in relation to gene expression levels within each subtype was not feasible, due to small sample sizes.

### Gene expression “heat maps” for 34 genes

Heat maps were constructed using 3 datasets from R2 (datasets 8, 9 and 10, Table 2). We aimed to display heterogeneity in the form of under- versus over-expression of 34 genes in the 4 GBM and 4 medulloblastoma subtypes (as defined in TCGA; Fig. 4 and Additional file 1: Figure S1, respectively), as well as in 4 types of pediatric brain tumors versus non-diseased brain (Additional file 2: Figure S2).

The heat maps were obtained by hierarchical clustering on samples within every defined subgroup of a dataset separately, followed by clustering over the genes (complete cohort).

## Results

### Differential expression of enzyme genes in GBM and proteins in HGG

Differential expression was defined as a ≥40% difference (higher or lower) in gene expression for any gene, in GBM compared to non-GBM specimens. Fewer than 30 genes involved in AA metabolism met this criterion (Fig. 1). Protein detection by IHC reflected gene expression levels in roughly two-thirds of the 34 genes (Fig. 2). Specifically, over-expressed genes had a higher proportion of samples with medium to high IHC staining of the expressed protein. In contrast, under-expressed genes were associated with low or undetected protein staining. This observation was true for most, but not all, genes and enzymes analyzed.

### Survival in relation to gene expression

**Fig. 3:**
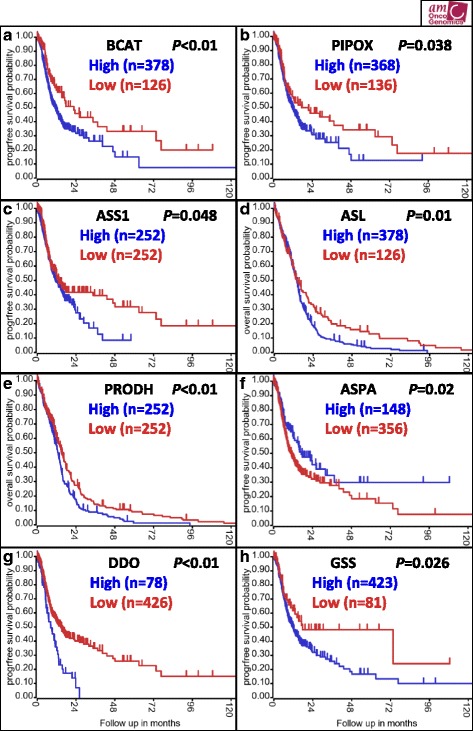
Representative Kaplan-Meier curves showing associations between expression of selected genes and patient survival. Gene names, numbers of samples with high versus low expression, and *P* values are shown in *boxes. X-axes* show follow-up in months, and *Y-axes* show survival probability. Panels **a**, **b**, **c**, **f**, **g**, and **h** show progression-free survival. Panels **d** and **e** show overall survival

Expression of some of the 34 genes correlated with progression free and/or overall survival (Fig. 3). For example, higher levels of some genes that are upregulated in GBM were associated with poor outcome, or via versa. However, other genes showed the reverse (occasionally following predictions based on protein levels). Some genes did not play a role in patient outcome altogether (Table 3). Interestingly, we also identified a group of genes that may play a role in outcome, but were not differentially expressed. Overall, it appears that dramatic differences in expression are more likely to result in survival differences, especially when gene expression correlates with protein production (Table 3). Genes that are over-expressed in GBM and also associated with poor survival at high expression levels may be the top candidates for therapeutic inhibition (dark gray shaded box in Table 3).

**Table 3:** Relationship between expressions of 34 selected genes and Kaplan-Meier analysis

	Enzymes for which …		
	higher expression is linked to poor survival	lower expression is linked to poor survival	expression is not correlated with survival
Enzymes with higher expression in GBM	**BCAT1** ^a^ ASL^a^ **LAP3** **PIPOX** ^a^ **GFPT2** **DDO** ^a^ FAH	DDAH2 **SHMT2** **TYMS** SHMT1	**TDO2** IDO1
Enzymes with expression as in normal brain	CNDP2 **GSS** ^a^ **GLUL (GS)**	PHGDH SEPHS1 **ABAT** ALT (GPT) **AGXT**	ASNS **MTR**
Enzymes with lower expression in GBM	**PRODH** ^a^ **ASS1** ^a^	AST (GOT1) **ASPA** ^a^ PAH	**GLUD1/GLUD2** GAD1/GAD2 **GLS** TH

^a^Survival curves for footnoted genes are shown in Fig. 3. Genes in bold have concordant protein (by IHC) and mRNA expression (by microarray)

### TCGA subtypes demonstrate heterogeneity for genes involved in AA metabolism

**Fig. 4:**
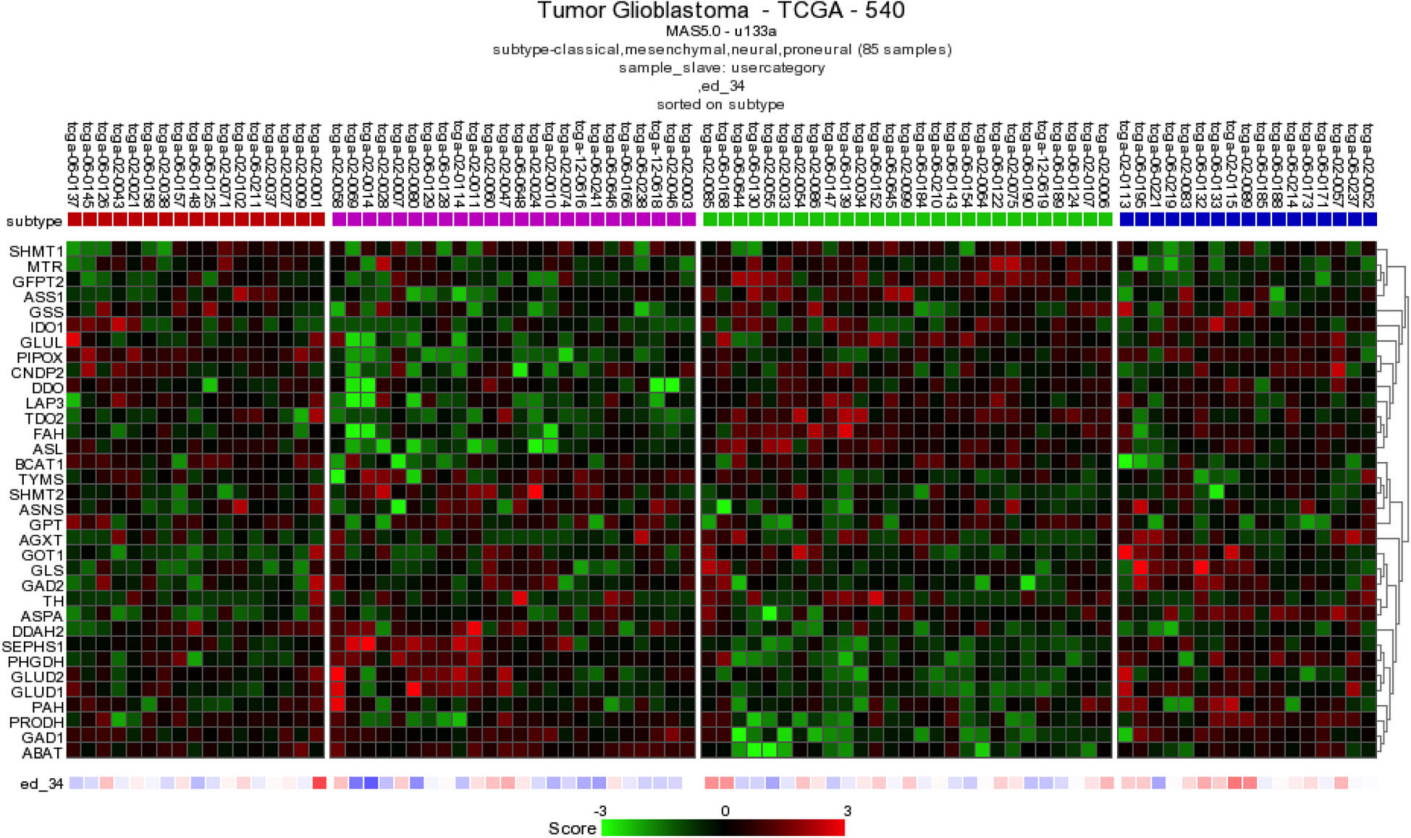
Heat map showing expression of 34 genes in GBM according to 4 TCGA subtypes. The *colored bars* at the tops of the heat maps indicate the GBM subtypes (from *left to right*): *red* – classical; *purple* – proneural; *green* – mesenchymal; *blue* – neural). Gene expression intensities are illustrated by *shades* of: *green* for lower levels of gene expression; *black* for a neutral level of gene expression; and *red* for higher levels of gene expression. Names of genes are abbreviated as in Table 1

Thirty-four genes were tested in one of the datasets, where TCGA grouping was available for 85 samples (17 neural, 17 classical, 27 mesenchymal and 24 proneural). A complex pattern of heterogeneity was observed (Fig. 4). Although further confirmation is needed, the results suggest distinct patterns of amino acid metabolism in the 4 TCGA subtypes, as measured by gene expression.

### Pediatric brain tumor types and medulloblastoma subtypes also may have distinct signatures of AA metabolism

In addition to GBM, we analyzed the same 34 genes in two other datasets in R2 (#9 and #10 in Table 2). One contains pediatric brain tumor samples (15 pilocytic astrocytomas, 34 glioblastomas, 22 medulloblastomas and 46 ependymomas). The other is a medulloblastoma dataset, grouped into 4 subtypes (10 SHH, 8 WNT, 16 G3, and 39 G4). As for GBM TCGA subtypes above (Fig. 4), we prepared gene expression heat maps reflecting over- and under-expression of genes in medulloblastoma subtypes and pediatric brain tumors (Additional file 1: Figure S1 and Additional file 2: Figure S2, respectively). In both cases, one can appreciate AA gene expression variability among the subtypes. There were no proteins or patient survival data available for analysis. However, these observations provide preliminary findings for further analysis and preclinical therapeutics development.

Findings on specific genes and enzymes are addressed in the Discussion section.

## Discussion

**Fig. 5:**
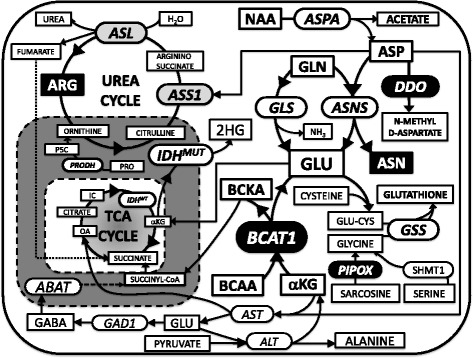
Summary of metabolic pathways in relation to selected potential targets for GBM therapy. The complex interplay among biochemical reactions in amino-acid metabolism in a metabolic network affects mitochondrial energy production and nitrogen utilization. Enzymes are in *rounded boxes*, and substrates are in *squared boxes*. A *few black boxes* highlight the most relevant targets. Abbreviations: ABAT, 4-aminobutyrate aminotransferase; αKG, alpha-keto-glutarate; ALT, alanine aminotransferase (also known as GPT); ARG, arginine; ASL, argininosuccinate lyase; ASN, asparagine; ASNS, asparagine synthetase; ASP, aspartate; ASPA, aspartoacylase; ASS1, argininosuccinate synthase 1; AST, aspartate aminotransferase (also known as GOT1); BCAAs, branched-chain amino acids; BCAT1, branched chain amino-acid aminotransferase 1; BCKA, branched chain ketoacids; CYS, cysteine; DDO, D-aspartate oxidase; GABA, gamma-amino butyric acid; GAD1, glutamate decarboxylase 1; GLN, glutamine; GLU, glutamate; GLS, glutaminase; GSS, glutathione synthetase; IC, isocitrate; IDH^MUT^ isocitrate dehydrogenase, mutated; 2HG, 2-hydroxyglutarate; NAA, N-acetyl-L-aspartic acid; OA, oxaloacetate; P5C, 1-pyrroline-5-carboxylate; PIPOX, pipecolic acid and sarcosine oxidase; PRO, proline; PRODH, proline dehydrogenase; SHMT1, serine hydroxymethyltransferase 1; TCA, tricarboxylic acid

Glioblastoma therapy continues to remain a major clinical challenge due to poor outcomes, with >90% of patients succumbing from their disease within 3 years of diagnosis [21]. Although immunotherapy and inhibition of cancer cell signaling hold promise, the “cornerstone” of current therapy against GBM remains DNA damaging strategies combined with surgery [22]. Targeting cancer metabolism by starving cancers of essential nutrients should be combinable with DNA damaging chemotherapy, due to lack of myelosuppression. Because lipid and energy metabolism is being investigated more intensively, this pilot study was designed to review brain tumor databases, to identify potentially druggable sites by interrogating amino acid-related metabolic pathways in GBM. Gene and protein expression patterns, in conjunction with survival data in GBM, were used as the main tools for searching for such targets. In addition, known amino acid depleting strategies, based on the available armamentarium and reported efficacy, are also considered in this discussion (Fig. 5). The analysis showed that 7 enzymes, namely, BCAT1, ASL, LAP3, PIPOX, GFPT2, DDO and FAH were upregulated variably in GBMs and were associated decreased survival. However, ASL and FAH upregulation did not translate into protein overproduction (Table 3 and Fig. 2). While it remains unclear how patient survival is affected by expression of these enzymes, a deeper follow-up metabolic exploration of brain cancers and other malignancies may be useful.

### *BCAT1* (branched chain amino acid transaminase 1)

The enzyme catalyzes the reversible transamination of branched-chain alpha-keto acids to branched-chain L-amino acids. BCAT1 has a well proven role in IDH^WT^ GBM reported in the literature [23]. In our study, there is higher expression of BCAT1 in GBM compared to non-GBM. Both PFS and OS are affected adversely by higher levels of expression in GBM, as well as by high levels of the protein (detected by IHC in HGGs). Taken together, these results suggest that development of BCAT1 inhibitors may have promising clinical potential. Neural and proneural tumors have lower BCAT1, making them less likely to respond to BCAA metabolism manipulation. The role of BCAT1 in other cancers may also be investigated.

### Arginine metabolism

Higher expression of ASS1 (argininosuccinate synthase 1) and ASL (argininosuccinate lyase) genes are associated with poor PFS and/or OS. However, only the ASL gene is differentially over-expressed in GBMs. And at the protein level, both ASL and ASS1 enzymes are low or undetected in HGGs. In spite of this complex pattern, it has been shown recently that human recombinant arginase-induced arginine depletion is selectively cytotoxic to human glioblastoma cells [24]. Moreover, arginine deiminase is active against GBM in vitro and in vivo [25]. Low ASS1 and ASL proteins in HGGs support further testing of arginine-depletion against GBM. An alternative formulation to be considered is PEG-ADI, which was used in a phase 2 trial for hepatocellular carcinoma [26].

Amino-acid depleting enzymes, such as arginase or asparaginase are large molecules, which may not penetrate an intact blood–brain barrier (BBB). Nevertheless, it is well documented that CSF asparagine, for instance, decreases significantly after asparaginase administration to acute lymphoblastic leukemia patients [27]. Therefore, penetration of these enzymes into parenchyma may not be necessary for an anti-tumor effect, inasmuch as substrate depletion influences the extra-vascular micro-environment of the CNS. In addition, parts of the BBB may not be completely intact [28] -- theoretically allowing direct entry of enzymes. Intracranial brain tumor mouse model testing will be the best next step to assess potential synergy of amino-acid depleting strategies with other therapies.

### Methionine

MTR (5-methyltetrahydrofolate-homocysteine methyltransferase) was the main methionine related enzyme, whose gene expression levels were slightly elevated in GBM. However, expression levels did not meet our definition of differential expression. MTR was not associated with clinical outcome. Moreover, there was neither differential expression in TCGA subtypes, nor high protein levels. Nevertheless, clinical observations, such as great diagnostic yields from 11C-MET PET uptake testing [29], support recently suggested research on methionine-free diets in combination with temozolomide against GBM (https://clinicaltrials.gov/ct2/show/NCT00508456). This study was terminated due to low accrual. Yet, preclinical research continues to support methionine deprivation as a potential therapy for GBM [30].

### Alanine and asparagine-glutamine networks

Some findings in these biochemical pathways can be summarized as differential under-expression of ASPA (aspartoacylase) and GOT1 (glutamic-oxaloacetic transaminase 1; previously known as AST, or aspartate aminotransferase) in GBM. Both are associated with poor outcome at lower gene levels, as is lower GPT (glutamic-pyruvic transaminase; previously known as ALT, or alanine aminotransferase). The neural group had higher GOT1 and ASPA gene expression, but lower GPT. Protein counterparts of GPT and GOT1 are overall more detectable in HGGs, compared to normal tissue, whereas ASPA protein is less detectable. ASPA catalyzes conversion of N-acetyl-L-aspartic acid (NAA) to acetate and is mutated in patients with Canavan disease. Detection of elevated NAA by magnetic resonance spectroscopy (MRS) is indicative of GBM progression. Some investigators have suggested that acetate supplementation (used for Canavan disease) may serve as an adjuvant therapy against GBM [31]. Acetate use against GBM may be supported by our findings of under-expression of the ASPA gene in GBM and the ASPA protein in HGGs. Acetate use is also supported by a strong signal from another over-expressed gene in our study -- PIPOX (pipecolic acid and sarcosine oxidase). PIPOX also shows high protein levels in HGGs, and high PIPOX is associated with poor outcome in GBM. PIPOX converts sarcosine to glycine (used by GSS, or glutathione synthetase) and can be inhibited by acetate [32].

The only individual, key-enzyme gene effect observed for glutamine metabolism in our study was for GLUL (glutamate-ammonia ligase; previously known as GS, or glutamine synthetase). Low GLUL levels correlated with better OS (Table 3). Nevertheless, a large body of literature suggests that the asparagine-glutamine node of amino acid metabolism may contain a credible potential target against GBM metabolism [33]. The combined effect of increased ASNS (asparagine synthetase), GLUL, and/or BCAT1 expression was shown in one of our recent studies to have a detrimental effect on patient outcomes [34]. Therefore, we consider and propose asparaginase/glutaminase as another potential adjuvant strategy against GBM. Differential expression of ASNS in ependymomas and certain types of medulloblastomas also supports asparaginase testing against these pediatric brain tumors.

### GABA metabolism

Mixed gene expression for GABA related enzymes indicated that decreased production and possibly increased catabolism may be linked to poor outcome. Gabapentin, a GABA analog, inhibits substance P-induced NF-kB activation in rat gliomas and may play role in regulating inflammation-related intracellular signaling [35]. However, the hypothesis of a significant antitumor effect of GABA against GBM remains unexplored, because its analogue, gabapentin (widely used in clinical practice without major anti-GBM effects), has no direct effect on GABA binding, uptake or degradation.

### Glutathione synthetase (GSS)

Interestingly, overexpression of the rate-limiting enzyme in glutathione synthesis (GCLM, or glutamate-cysteine ligase modifier subunit) was not detected in these analyses. Likewise, GSS levels were not much altered at baseline. One may predict that a potential role of GSS inhibition by the available agent, buthionine sulfoximine (BSO), may be limited to chemotherapy-induced, GSS-up-regulation cases. This has been a subject of significant research for other cancers, but not GBM [36]. A study to assess GSS upregulation after chemotherapy in GBM may be useful. Analysis of enzymatic and non-enzymatic components of antioxidant pathways -- apart from amino-acid metabolism -- is another valid topic for study.

### Tryptophan

IDO1 (indoleamine 2, 3-dioxygenase 1) catalyzes tryptophan breakdown. Its inhibitors are aimed at suppressing tryptophan catabolism-induced cancer immunotolerance and are in clinical trials (https://www.clinicaltrials.gov/show/NCT02052648). No survival link or differential expression was observed in our analysis for GBM versus non-GBM brain tissues for IDO1 or TDO2 (tryptophan 2, 3-dioxygenase, also involved in tryptophan catabolism). However, our findings showed higher TDO2 and IDO1 in GBM, and particularly in the mesenchymal subtype, which may show better responses to immunotherapy [37]. These reports further support a potential role for manipulating tryptophan metabolism for cancer immunomodulation effects [30, 38].

### Other genes

Potential targets can be expanded to a few other important genes based on our results, including: GFPT2 (glutamine-fructose-6-phosphate transaminase 2; previously reported to be high in GBM [39]); LAP3 (leucine aminopeptidase); DDO (D-aspartate oxidase); and PRODH (proline dehydrogenase, a putative tumor suppressor). Retrospective studies and preclinical validations are needed, because gene and protein databases used in this study are not the same. Also, no protein data were available on pediatric tumors and medulloblastoma. Furthermore, changes may occur in response to chemo/radiation treatments, and the tumors may harbor unknown mutations in some of these pathways (a possible subject of future studies).

## Conclusions

Brain tumors have distinct gene expression patterns for certain amino acid-metabolizing enzymes. These enzymes may provide valid targets for therapeutics development. Although drugs used clinically, such as asparaginase and arginase, are readily available for preclinical testing, inhibitors have yet to be developed against other promising targets, such as BCAT1 or PIPOX. Heterogeneity is evident in various types (and subtypes) of brain tumors, which indicates the possible need for tailored manipulation of amino acid metabolism to achieve enhanced therapeutic effects and less toxicity than encountered with conventional chemotherapy.

## Additional files

Additional file 1: Figure S1. Heat map of expression of 34 genes in 4 subtypes of medulloblastoma -- WNT, SHH, Group 4 and Group 3. Names of genes are abbreviated as in Table 1. (DOCX 92 kb)
Additional file 2: Figure S2. Heat map of expression of 34 genes in 4 types of pediatric brain tumors and non-diseased (nd) brain tissue (from left to right: glioblastomas, nd, ependymomas, medulloblastomas and pilocytic astrocytomas). Names of genes are abbreviated as in Table 1. (DOCX 111 kb)

## Abbreviations

2HG: 2-hydroxyglutarate
AA: Amino-acids
ABAT: 4-aminobutyrate aminotransferase
ARG: Arginine
ASL: Argininosuccinate lyase
ASN: Asparagine
ASNS: Asparagine synthetase
ASP: Aspartate
ASPA: Aspartoacylase
ASS1: Argininosuccinate synthase 1
BBB: Blood–brain barrier
BCAAs: Branched-chain amino acids
BCAT1: Branched chain amino acid transaminase 1
BCKA: Branched chain ketoacids
DDO: D-aspartate oxidase
GABA: Gamma-amino butyric acid
GAD1: Glutamate decarboxylase 1
GAD2: Glutamate decarboxylase 2
GBM: Glioblastoma
GFPT2: Glutamine-fructose-6-phosphate transaminase 2
GLN: Glutamine
GLS: Glutaminase
GLU: Glutamate
GLUL: Glutamate-ammonia ligase
GOT1: Glutamic-oxaloacetic transaminase 1
GPT: Glutamic-pyruvic transaminase
GSS: Glutathione synthetase
IC: Isocitrate
IDH^MUT^: Isocitrate dehydrogenase, mutated
IDH^WT^: Isocitrate dehydrogenase, wild type
IDO1: Indoleamine 2,3-dioxygenase 1
KEGG: Kyoto Encyclopedia of Genes and Genomes
LAP3: Leucine aminopeptidase
MRS: Magnetic resonance spectroscopy
MTR: 5-methyltetrahydrofolate-homocysteine methyltransferase
NAA: N-acetyl-L-aspartic acid
NFkB: Transcription factor complex nuclear factor-kappa-B
OA: Oxaloacetate
OS: Overall survival
PFS: Progression-free survival
PIPOX: Pipecolic acid and sarcosine oxidase
PRO: Proline
PRODH: Proline dehydrogenase
PRODH: Proline dehydrogenase
TCGA: The Cancer Genome Atlas
TDO2: Tryptophan 2,3-dioxygenase
