# Supplementary figures and images for: In search of druggable targets for GBM amino acid metabolism
Source: BMC Cancer. 2017 Feb 28;17:162. doi: 10.1186/s12885-017-3148-1 (PMC5331648; doi:10.1186/s12885-017-3148-1)

**Figure S1.** Heterogeneity of 34 enzymes in 4 subtypes of Medulloblastoma


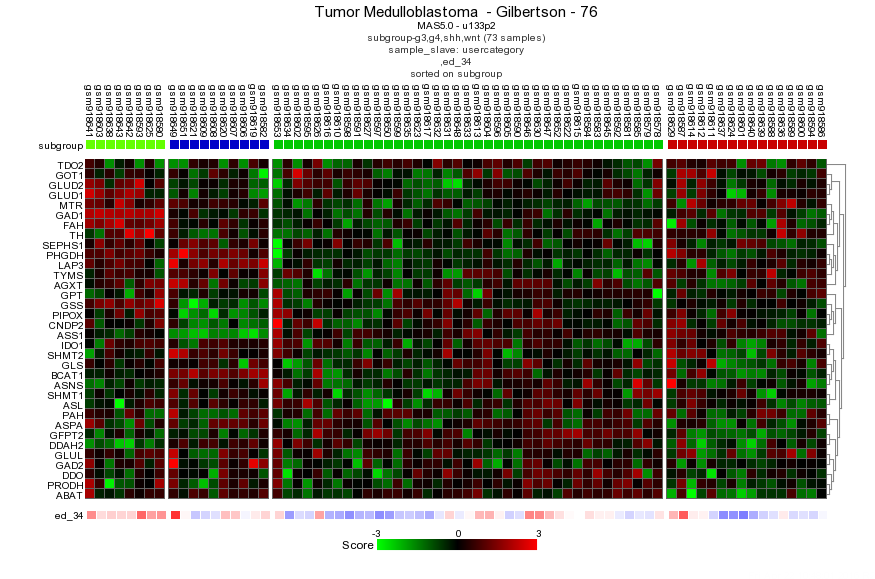

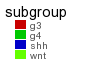

Supplement: Additional file 1: Figure S1. — Heat map of expression of 34 genes in 4 subtypes of medulloblastoma -- WNT, SHH, Group 4 and Group 3. Names of genes are abbreviated as in Table 1. (DOCX 92 kb) [file 12885_2017_3148_MOESM1_ESM.docx]

**Figure S2.** Heterogeneity of 34 enzymes in pediatric brain tumors and not-diseased (nd) brain tissue


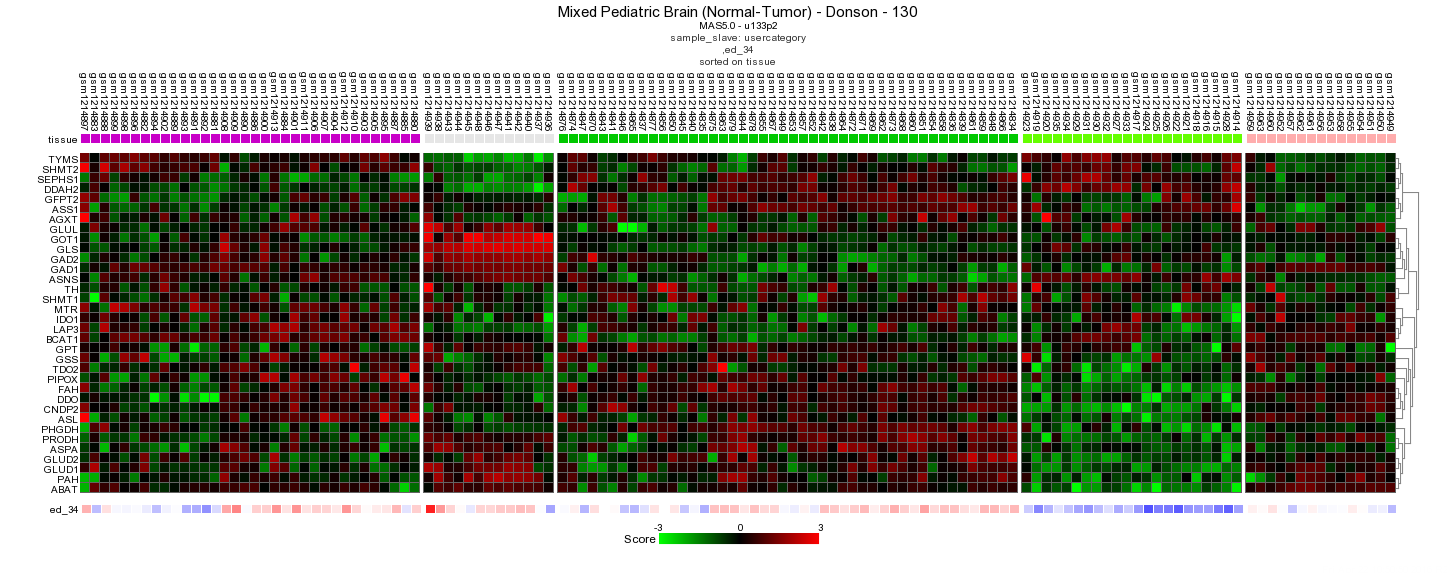


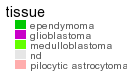

Supplement: Additional file 2: Figure S2. — Heat map of expression of 34 genes in 4 types of pediatric brain tumors and non-diseased (nd) brain tissue (from left to right: glioblastomas, nd, ependymomas, medulloblastomas and pilocytic astrocytomas). Names of genes are abbreviated as in Table 1. (DOCX 111 kb) [file 12885_2017_3148_MOESM2_ESM.docx]
